# Neural Dynamics of Visual Stream Interactions During Memory-Guided Actions Investigated by Intracranial EEG

**DOI:** 10.1007/s12264-025-01371-x

**Published:** 2025-03-17

**Authors:** Sofiia Moraresku, Jiri Hammer, Vasileios Dimakopoulos, Michaela Kajsova, Radek Janca, Petr Jezdik, Adam Kalina, Petr Marusic, Kamil Vlcek

**Affiliations:** 1 Laboratory of Neurophysiology of Memory, Institute of Physiology, Czech Academy of Sciences, Prague, Czechia; 2 Third Faculty of Medicine, Charles University, Prague, Czechia; 3 Department of Neurology, Second Faculty of Medicine, Charles University and Motol University Hospital, Member of the Epilepsy Research Centre Prague - EpiReC consortium, Prague, Czechia; 4 Department of Circuit Theory, Faculty of Electrical Engineering, Czech Technical University in Prague, Member of the Epilepsy Research Centre Prague - EpiReC Consortium, Prague, Czechia; 5 Klinik für Neurochirurgie, Universitätsspital Zürich, Universität Zürich, Zurich, Switzerland

**Keywords:** Dorsal visual stream, Ventral visual stream, Memory-guided actions, Intracranial EEG, Phase-locking value, Granger causality analysis, Alpha oscillations, Theta oscillations

## Abstract

The dorsal and ventral visual streams have been considered to play distinct roles in visual processing for action: the dorsal stream is assumed to support real-time actions, while the ventral stream facilitates memory-guided actions. However, recent evidence suggests a more integrated function of these streams. We investigated the neural dynamics and functional connectivity between them during memory-guided actions using intracranial EEG. We tracked neural activity in the inferior parietal lobule in the dorsal stream, and the ventral temporal cortex in the ventral stream as well as the hippocampus during a delayed action task involving object identity and location memory. We found increased alpha power in both streams during the delay, indicating their role in maintaining spatial visual information. In addition, we recorded increased alpha power in the hippocampus during the delay, but only when both object identity and location needed to be remembered. We also recorded an increase in theta band phase synchronization between the inferior parietal lobule and ventral temporal cortex and between the inferior parietal lobule and hippocampus during the encoding and delay. Granger causality analysis indicated dynamic and frequency-specific directional interactions among the inferior parietal lobule, ventral temporal cortex, and hippocampus that varied across task phases. Our study provides unique electrophysiological evidence for close interactions between dorsal and ventral streams, supporting an integrated processing model in which both streams contribute to memory-guided actions.

**Supplementary Information:**

The online version contains supplementary material available at 10.1007/s12264-025-01371-x.

## Introduction

There has been an extensive debate on the precise role of the dorsal and ventral visual streams in visual processing for action. The original perception-action model, proposed by Goodale and Milner [1], implies that the dorsal (in the occipito-parietal cortex) and ventral (in the occipito-temporal cortex) streams process visual information for different purposes: for motoric action and for conscious perception, respectively. The model also suggests that the two streams differ in storage capacity [2, 3]: the dorsal stream operates in real-time, storing visual information briefly for immediate action guidance, while the ventral stream appears to facilitate memory-guided or delayed actions. When visual information for action must be kept in memory for several seconds [2], the ventral stream becomes crucial for guiding the visuomotor act. Memory-guided actions rely on working memory, which is responsible for temporarily holding and manipulating information over short periods [4], and require the integration of sensory information, memory processes, and motor planning to execute actions based on previously encountered stimuli [2]. Therefore, investigating the neural mechanisms underlying memory-guided actions inherently involves studying working memory processes.

Evidence supporting the transient nature of the dorsal stream storage capacity is based on studies of brain-damaged patients. For instance, patient D.F. with bilateral ventral lesions in the lateral occipital complex, demonstrated impaired object grasping after a short delay [3]. Conversely, patients with parietal lobe lesions and optic ataxia were less impaired after a short delay of 5 s compared to no delay [5], suggesting compensation by stored information in the ventral stream. Previous fMRI studies have also implied that ventral stream regions play a role in delayed reaching and grasping [6], and transcranial magnetic stimulation inhibition of the lateral occipital complex in the ventral stream disrupted only delayed but not immediate grasping [7].

However, accumulating evidence suggests that the dorsal stream, particularly the parietal cortex, is also involved in working memory maintenance and motor planning. The parietal cortex has been shown to be involved in the maintenance of various visual short-term memories [8–10]. Neuroimaging studies have demonstrated sustained activity in the parietal lobe, particularly the inferior parietal lobule (IPL), during the delay periods of working memory action tasks, suggesting its role in maintaining target information for delayed reaching and grasping [11, 12]. These findings challenge the original view that the dorsal stream is exclusively involved in immediate action guidance, demonstrating that it also contributes to working memory processes necessary for memory-guided actions. In line with these findings, Schenk and Hesse [13] argue that the ‘dorsal amnesia hypothesis’ oversimplifies the complex interactions between the dorsal and ventral streams in guiding actions. Their review, along with other studies, supports the view that both streams contribute to working memory and motor planning and that their interactions might be crucial for memory-guided actions.

Despite the evidence for the dorsal stream's involvement in working memory and motor planning, the exact nature and directionality of the interactions between the dorsal and ventral streams during memory-guided actions remain to be fully elucidated. Understanding not just whether, but how information flows between these regions is crucial for clarifying their roles in complex cognitive tasks involving working memory. Electrophysiological studies can provide valuable insights into the temporal dynamics and functional connectivity between these regions, including the direction of information transfer. Moreover, previous studies have not fully addressed how the two visual streams interact during the maintenance of visuospatial information, especially when processing identical *versus* different objects for delayed actions. The hippocampus may play an important role in processing related objects, particularly when both identity and location information are maintained, as has been implied in the maintenance of various features in episodic and working memory [14–16]. During this process, the hippocampus may interact with the dorsal stream to support the organization and utilization of position and identity information for memory-guided actions.

To further elucidate the roles of the dorsal and ventral streams, as well as the hippocampus, and their interactions during memory-guided actions we used intracranial EEG (iEEG) to investigate the dynamics and directional flow of communication between these regions. The high temporal resolution of iEEG allows for tracking neural activity simultaneously in the brain regions of the dorsal (IPL) and ventral (ventral temporal cortex, VTC) streams, as well as in the hippocampus, and studying their interactions *via* functional connectivity measures, including directional connectivity using Granger causality analysis. We focused on low-frequency theta and alpha activities due to their roles in working memory [8, 10, 17, 18]. We analyzed functional connectivity using the phase-locking value (PLV) between dorsal and ventral stream areas, as synchronized oscillations have been proposed as a mechanism for functional interactions between brain regions [19, 20]. These oscillations are considered to mediate long-range communication between cortical areas by low-frequency phase coupling [17, 21, 22]. To assess the directionality of these interactions, we applied Granger causality analysis, which can determine the influence of one brain region on another over time [23, 24].

Our study involved nine patients with drug-resistant epilepsy undergoing iEEG monitoring. They performed a delayed action task in a paradigm with two objects and a central cross. Their goal was to estimate which object was closer to the cross and to reach for it with a joystick after a jittered 4-s delay. This task required participants to hold visuospatial information in working memory over a 4-s delay and then execute a motor response based on that information, thus engaging both working memory processes and memory-guided action planning. We used two conditions: "same objects" and "different objects", thus requiring attention to both object identity and location. Without any delay, this task is a simple egocentric distance estimation that primarily activates the parietal lobe in the dorsal stream (see review [25]). However, in the delayed task, we hypothesized the following: (1) sustained low-frequency activity in both the dorsal (IPL) and ventral (VTC) streams during the delay, based on the new framework of their combined role in processing visual information for memory-guided actions [13] and in working memory maintenance; (2) increased phase synchronization between the IPL and VTC during the delay in both conditions, indicating task-dependent interactions of spatial information across streams; (3) additional activity in the hippocampus and stronger IPL-hippocampus phase synchronization during the delay in the "different objects" condition, supporting the maintenance of object identity and position information; and (4) directional influences between these regions that vary across task periods, with the ventral stream (VTC) exerting greater influence on the dorsal stream (IPL) during the later stages of the delay in preparation for the action, and the IPL influencing the hippocampus during memory maintenance.

By addressing these hypotheses, our study aimed to advance the understanding of visual information processing in the brain, explore the neural basis of memory-guided actions within the context of working memory, and further clarify the interactions and directional influences between the dorsal and ventral streams and the hippocampus.

## Materials and Methods

### Patients

Nine patients with drug-resistant epilepsy (4 women; mean (± SEM) age = 37 ± 3 years, see Table S1 for details) were enrolled at the Motol Epilepsy Center in Prague. They underwent iEEG monitoring for precise localization of the epileptic seizure onset zone before surgery. All the patients signed informed consent to participate, and the study was approved by the Ethics Committee of Motol University Hospital. All the patients had normal or corrected-to-normal vision.

### Behavioral Task

**Fig. 1:**
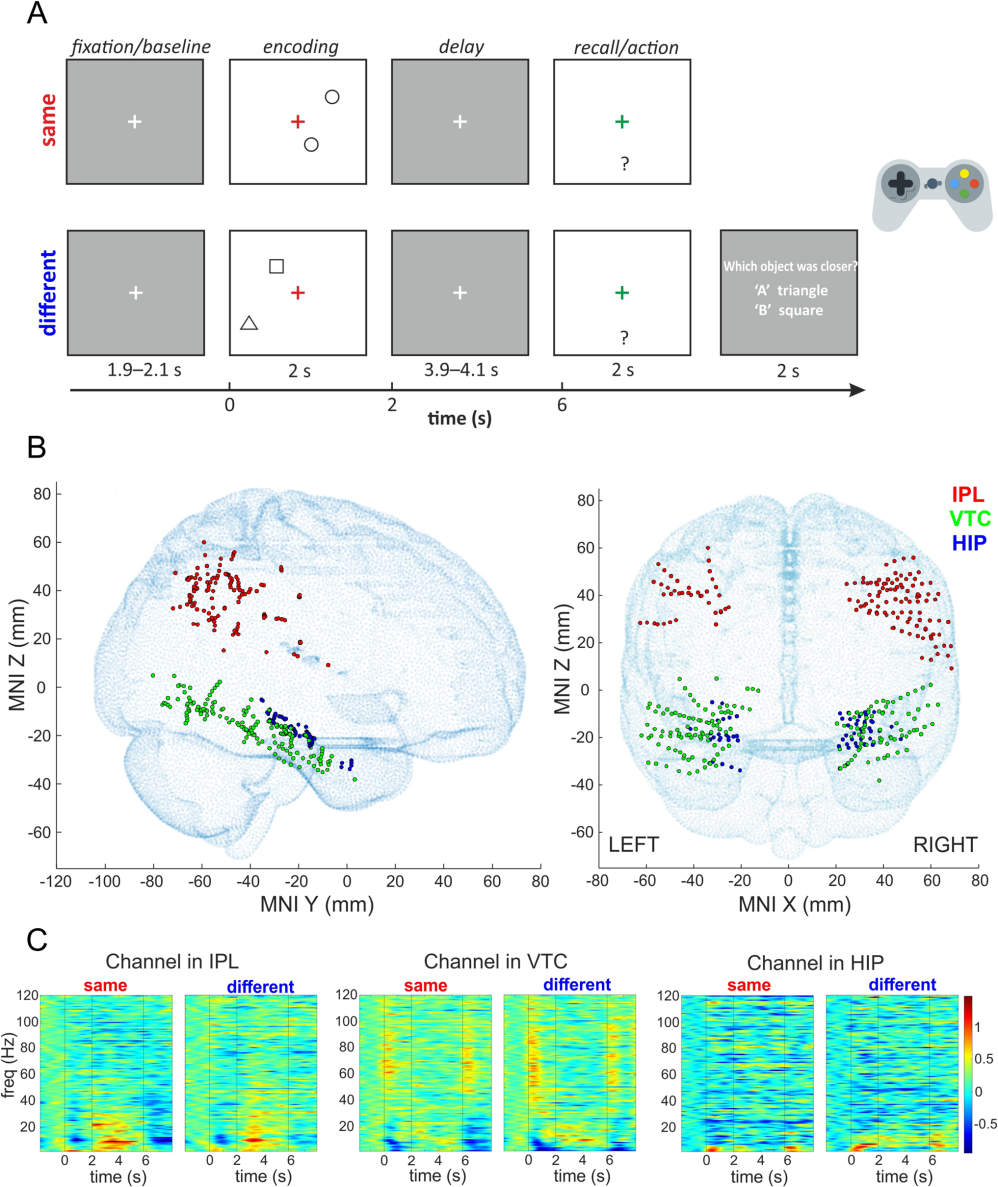
Task design, iEEG channel locations, and exemplary responses. **A** Experimental design of the delayed action task. Each trial includes a jittered fixation period (1.9 s–2.1 s), encoding (2 s), a jittered delay (3.9 s–4.1 s), and an action/recall phase (2 s). Participants have to remember the object's position (same condition) or both its position and identity (different condition) and reach for it with a joystick after the delay. In the different condition, a two alternative question about the object's identity is followed with a 2 s time-window to answer. Participants respond using the gamepad: green 'A' for a triangle or red 'B' for a square. **B** The projection of all implanted channels in 3 brain regions in 9 patients (369 channels) from both hemispheres on the standard MNI brain template (adult MNI-ICBM152 head model, [26]; http://www.ucl.ac.uk/dot-hub). Left, sagittal view; right, coronal view. IPL, inferior parietal lobule; VTC, ventral temporal cortex; HIP, hippocampus. **C** Trial-averaged spectrograms of exemplary individual channels in IPL, VTC, and HIP for the same and different conditions, 0—the start of the encoding phase; vertical lines mark the boundaries of the task periods, and only the last 3.9 s of delay are plotted. Prominent changes during the delay occur at low-frequency power.

The experimental task aimed to investigate the neural processes involved in memory-guided actions based on different types of visual information. Each trial consisted of fixation, encoding, delay, and action/recall phases. The trial began with a jittered fixation period of 1.9–2.1 s, displaying a white cross on a dark gray screen (Fig. 1A), followed by a 2 s encoding period with a central red cross and two types of objects: either two identical circles ("same" condition) or a square and a triangle ("different" condition). The positions of the objects were randomized for each trial, with one object always positioned closer to the cross than the other, maintaining a consistent distance ratio of 1.5. In the 'different' condition, the triangle was closer in 50% of the trials, and the square was closer in the other 50%. The participant's task was to remember which object was closer to the cross: its position in the 'same' condition and both its position and identity in the 'different' condition. Since the cross was always centered on the screen, corresponding to the participant's midsagittal plane, we assume that distance estimation was performed using egocentric coordinates.

After the encoding phase, a white fixation cross reappeared on a dark gray screen during a delay phase, jittered between 3.9 s and 4.1 s to prevent anticipatory responses. Similar jitter ranges have been effectively used in previous studies [9, 10]. Although this range may not entirely eliminate anticipation, it was selected for task feasibility and participants’ comfort, given the clinical settings of our experiment. Following this, a green cross appeared for 2 s during the action/recall phase, prompting participants to respond. Using a gamepad joystick (Xbox wireless controller), they moved the green cross to the memorized position of the nearest object. The subjects made a straight reach from the center of the screen to the memorized object and returned to the center of the screen. A response was considered correct if the minimum distance of their trajectory was within a square area defined as the X,Y coordinates of the correct object plus or minus half the minimum distance between objects over all trials (10.5% of the screen width). Reaction time was measured from the start of the action phase to the time of reaching the correct area. In the ‘different' condition, an additional two-alternative question about the object's identity appeared for 2 s at the end of the trial. Participants responded by pressing a button on the gamepad: the green 'A' button for a triangle or the red 'B' button for a square. The total trial duration, including the pre-trial period, ranged from 9.8 s to 10.2 s for the 'same' condition and from 11.8 s to 12.2 s for the 'different' condition.

The task was counterbalanced with 160 trials equally divided between the 'same' and 'different' conditions. Trials were grouped into blocks of 10, each assigned to one condition, with a subject-controlled break between blocks. The order of blocks was counterbalanced so that the average order of 'same' and 'different' blocks was the same. At the beginning of each block, participants received simple on-screen instructions about the upcoming condition. The experiment began with a brief presentation explaining the task, followed by a training session with shortened blocks of five trials per condition, with feedback provided after each trial. The experiment also included 160 immediate trials, in which participants reached for the object immediately without any delay, again equally divided between the 'same' and 'different' conditions. As the main focus of this study was the analysis of the delayed trials, in particular the functional connectivity between the two streams during the maintenance phase, we report the results of the analysis of the immediate trials in the Supplementary Materials.

The experimental task was implemented in PsychoPy3 v2020.1.3 [27] and run on a 15.6-inch TFT notebook monitor with a refresh rate of 60 Hz. The task and iEEG recording were synchronized by TTL pulses sent to a trigger port of the iEEG recording system at the start of each trial.

### Intracranial EEG Recording and Preprocessing

The iEEG was recorded with stereotactically implanted multi-contact electrodes, often also referred to as stereo-EEG. Recording sites were selected on an individual basis, strictly according to the medical requirements of the pre-surgical evaluation of epileptic zones, without reference to the present study. Eleven to fifteen semi-rigid electrodes were implanted intracerebrally per patient, depending on the suspected origin of their seizures. Each electrode had a diameter of 0.8 mm and consisted of 8–18 contacts, 2 mm long, and 1.5 mm apart (DIXI Medical Instruments). The iEEG signal was recorded with medical amplifiers (Quantum, NeuroWorks), sampled at 2048 Hz, and later downsampled to 512 Hz to reduce the computational load. The reference contact for each patient was located in the white matter. Post-implantation CT, co-registered with pre-implantation MRI, was used to identify the positions of electrode contacts in each patient [28]. The anatomical locations of the electrode contacts were labeled by an experienced neurologist. The contact positions were then normalized to the Montreal Neurological Institute (MNI) space using standard Statistical Parametric Mapping algorithms (SPM 12) for group-level visualizations.

The downsampled iEEG recordings were first visually inspected, and any bad electrode contacts with obvious artifacts were discarded. Contacts identified as being in the seizure onset zone or heterotopic cortex were also excluded. Bipolar derivations were calculated between adjacent contacts to suppress contributions from distant neuronal assemblies and enhance spatial specificity. Importantly, bipolar re-referencing reduces the effects of volume conduction and common reference artifacts, which can lead to spurious correlations in connectivity analyses due to shared signals [23, 29]. Bipolar iEEG signals were visualized at the center between two contacts. Henceforth, we refer to the bipolar contact pair simply as a 'channel'. When the channel was derived from two contacts in a different brain structure, we labeled it with the structure with a larger unilateral response. Line noise was removed from the iEEG signal with a notch filter (4th order Butterworth stop-band filter of 1 Hz width centered at 50 Hz and harmonics, zero phase shift). Data processing and analysis were performed in MatLab R2018a.

For further analysis, we selected channels located in three brain regions of interest (containing a total of 369 channels in 9 patients, see Table 1 and Fig. 1B): the IPL, including supramarginal and angular gyri and the adjacent lateral wall of the intraparietal sulcus; the VTC, including the inferior temporal, lingual, fusiform and parahippocampal gyri; and the hippocampus (HIP).

**Table 1:** Number of implanted and active channels in each brain region

Brain region	Total number of patients	Total number of channels	Number of channels active in the alpha band (N patients)	Number of channels active in the theta band (N patients)
IPL	8	137	44 (7)	31 (7)
VTC	9	169	64 (8)	29 (6)
HIP	8	63	20 (7)	18 (5)

### Time-Frequency Analysis

Time-frequency analysis was applied for linearly increasing frequencies between 2 and 120 Hz, with a resolution of 1 Hz bins, using a technique called the filter-Hilbert method. Similar to our previous studies [30–32], we estimated the power change using the following procedure. First, we band-pass filtered the entire recording dataset (third-order Butterworth filter, zero phase shift) in consecutive non-overlapping 1 Hz frequency bands. For each band, we extracted the amplitude envelope using the Hilbert transform; the obtained envelope was downsampled to 64 Hz, resulting in a time resolution of 15.625 ms. The envelope of each band was normalized by dividing it by its mean value over the entire recording session, channel-wise for each frequency band, effectively whitening the broad frequency band and compensating for the 1/f frequency decay of EEG signals [33]. Then, similar to [10] and based on visual inspection of time-frequency spectrograms, we extracted power for two non-overlapping frequency bands: theta (2–7 Hz) and alpha (8–13 Hz). To do this, the original 1 Hz bands in these theta and alpha ranges were averaged and multiplied by 100 to obtain a single time series of theta and alpha power for each channel expressed as percentages of the mean value. These two-time series signals were then divided into epochs. To ensure that the epochs had the same duration, we left out from the epochs the jittered period from the beginning of the delay phase (0.0s–0.2s). Each epoch was then divided into the following five task periods: (1) baseline (0.5 s, end of the fixation period), (2) encoding (2.0 s), (3) first half of delay (1.9 s, referred to as Delay 1 in the text), (4) second half of delay (1.9 s, referred to as Delay 2) and (5) recall (2.0 s). In the result, the epochs were from −500 ms to 7800 ms relative to the stimulus onset - the beginning of the encoding phase.

The mean of the −500 ms to 0 ms of the fixation period (i.e. the baseline) was subtracted from each epoch to remove signal changes independent of the respective stimulus. We excluded from further analysis epochs in each channel containing interictal epileptiform discharges, which were identified by a spike detector implemented in MatLab [34]. In addition, trials with incorrect behavioral responses—incorrect joystick responses for the object's position and incorrect button responses for the object's identity—were excluded from the iEEG analysis.

To test for the significance of the mean power in a given frequency band relative to the baseline, we used a Wilcoxon signed-rank test, with false discovery rate (FDR) correction across the time samples and channels, similar to our previous studies [30–32]. For each channel, we compared the average power for all trials of the respective condition during the pre-stimulus interval (–500 ms–0 ms before encoding) with all the time points during the post-stimulus period (0 ms–7800 ms). As a conservative estimate, we used a sliding window of six samples (93.75 ms) with the highest *p*-value. If there was a significant difference at any time point relative to the baseline for a selected condition, the channel was considered 'active' in the given frequency band.

Then, for all active channels in a given frequency band (theta or alpha), we averaged the power in each task period: encoding, first and second halves of delay, and recall. These averaged values were submitted to a linear mixed effects model (LMEM) with task period and condition and their interaction as fixed effects and channel and patient as random effects (*P <*0.008, Bonferroni correction for six models - three brain regions and two frequency bands):

$$ {\text{meanPower }}\sim { 1 } + {\text{ taskPeriod }}*{\text{ condition }} + \, \left( {{1 }|{\text{ patient}}} \right) \, + \, \left( {{1 }|{\text{ patient}}:{\text{channel}}} \right) $$

### Phase-Locking Value (PLV) Analysis

To evaluate the functional connectivity between channel pairs, we calculated the PLV [35]. This was done using a multitaper frequency transformation with two tapers based on the Fourier transform, covering a frequency range of 2–20 Hz with a resolution of 1 Hz, as implemented in the FieldTrip toolbox [36], and similar to other studies [17, 24].

The PLV between channels i and j is defined as:

$$ {\text{PLV}}_{ij} \left( f \right) = \frac{1}{N}\left| { \mathop \sum \limits_{n = 1}^{N} \frac{{X_{i } \left( f \right) \cdot \left( {X_{j} \left( f \right)} \right)^{* } }}{{\left| {X_{i} \left( f \right)} \right| \cdot \left| {X_{j} \left( f \right)} \right|}} } \right| $$

where N is the number of trials, X(f) is the Fourier transform of signal x(t) (1.9 s task period), and (∙)* represents the complex conjugate. Note that this formula is equivalent to the PLV calculation presented by Lachaux *et al*. [35] and should not be confused with the coherence equation.

Phase differences were calculated for each channel pair (i,j) between IPL and VTC, and between IPL and HIP, using the spectra of the encoding, the two halves of the delay, and the recall periods to quantify inter-electrode phase coupling. To determine statistically significant differences in the PLV during the encoding, both parts of the delay, and the recall, we compared them to the baseline (1.9 s fixation interval) using permutation statistics with cluster correction. The null distribution was created by calculating the PLV differences between the baseline and each task period (the encoding, the first half of the delay (1.9 s, Delay 1), the second half of the delay (1.9 s, Delay 2), and the recall (0.5 s, the part before the response was made)) for randomized data. Trials were randomly assigned to these two periods, and the PLV differences between them were calculated and repeated 200 times. This randomization preserves any frequency-dependent biases due to temporal smoothness present in both task and baseline periods. Only those frequency bins with PLV above the 95th percentile threshold of the null distribution were considered statistically significant [17, 24]. Cluster correction was applied to account for multiple comparisons across the 2–20 Hz frequency range (*P <*0.05 was used to obtain null-hypothesis clusters). A channel pair was considered significant if its PLV was significant at any frequency point in the 2–20 Hz range after cluster correction.

We found that different channel pairs exhibited PLV significance at various frequency points within the 2–20 Hz range. We aggregated all channel pairs significant in any of the four task periods (the encoding, two parts of the delay, and recall) from all patients and calculated the ratio of significant pairs (Sig.P.Ratio) in each 1-Hz frequency bin. This was done by dividing the number of significant pairs between two brain regions by the total number of channel pairs between these regions, for all subjects and in each 1-Hz bin independently [37]. This Sig.P.Ratio was calculated separately for IPL-VTC and IPL-HIP pairs. To identify frequency ranges at which the proportion of channel pairs was significantly above the chance level, we applied the binomial test to these Sig.P.Ratio values (one-sided test, *P <*0.05, FDR-corrected across frequency bins). The median Sig.P.Ratio of each region pair across all frequency bins was used as the chance level for the binomial test [37].

We then averaged the PLV for each task period (baseline, encoding, two parts of the delay, and recall) for each frequency range with a Sig.P.Ratio significantly higher than the median chance level. These averaged values were then analyzed using the LMEM with task period, condition, and their interaction as fixed effects, and channel pair and patient as random effects (*P* <0.0167, Bonferroni correction for three models, each for one identified frequency range; see Results):

$$ {\text{meanPLV }}\sim { 1 } + {\text{ taskPeriod }}*{\text{ condition }} + \, \left( {{1 }|{\text{ patient}}} \right) \, + \, \left( {{1 }|{\text{ patient}}:{\text{channelPair}}} \right) $$

### Spectral Granger Causality Analysis

To evaluate the direction of information flow between IPL and VTC, and between IPL and HIP, we applied spectral non-parametric Granger causality (GC) analysis as a measure of directed functional connectivity [23]. Similar to another study [24], we downsampled the iEEG signals to a sampling rate of 40 Hz and calculated the spectral non-parametric GC in the 1–20 Hz frequency range. We transformed the signals into the frequency domain using the multitaper method with two Hann tapers and zero-padding to 20 s to reduce spectral leakage and control frequency smoothing.

In the frequency domain, GC quantifies the extent to which the spectral content of the source signal contributes to the spectral content of the target signal at each frequency. We used a non-parametric spectral approach to compute GC at a given interval time [23]. In this method, the spectral transfer function H(f) and the noise covariance matrix Σ are estimated from the cross-spectral density matrix obtained from the Fourier transforms of the data. The total power spectral density matrix S(f) is computed as S(f) = H(f)ΣH*(f), where H*(f) denotes the complex conjugate transpose of H(f). The GC from signal Y to signal X at frequency f is then calculated as:

$$ {\text{GC}}_{Y \to X} \left( f \right) = \ln \left. {\left( {\frac{{S_{xx } \left( f \right)}}{{\tilde{S}_{xx } \left( f \right)}}} \right.} \right) $$

Where S_*xx*_(f) is the total power of signal X, and S̃_*xx*_(f) is the intrinsic power of X at frequency f when the influence of Y is removed.

We applied the GC analysis using the implementation in the FieldTrip toolbox [36]. GC was calculated for four task periods—baseline (fixation), encoding, delay 1, and delay 2 (each 1.9 s in duration)—for each condition (same and different objects). We did not analyze the recall phase because it was too short for reliable spectral GC analysis; behavioral responses started ~500 ms–600 ms after the beginning of the recall phase, making the effective time window insufficient for accurate spectral estimation. We focused our analysis on the channel pairs between IPL and VTC, and between IPL and HIP, which were found to be significant in the PLV analysis, as these pairs already suggested meaningful interactions. By running the GC analysis only on these pairs, we concentrated on the most relevant connections.

To identify the frequency ranges where the difference in directional influence between the two directions in VTC-IPL connections was the largest, we aggregated the GC data across all subjects, channel pairs, task periods, and conditions by averaging, and computing the difference in GC values (net Granger, similar to [24, 38]) between the two directions for each frequency bin:

$$\Delta {\text{GC}}_{{{\text{VTC }} \to {\text{IPL}}}} = {\text{ GC}}_{{{\text{VTC }} \to {\text{IPL}}}} - {\text{ GC}}_{{{\text{IPL }} \to {\text{VTC}}}} $$

and similarly for HIP and IPL:

$$\Delta {\text{GC}}_{{{\text{HIP }} \to {\text{IPL}}}} = {\text{ GC}}_{{{\text{HIP }} \to {\text{IPL}}}} - {\text{ GC}}_{{{\text{IPL }} \to {\text{HIP}}}} $$

We identified the frequency ranges where the net GC showed maximal differences. We then averaged the net GC over these frequency ranges for each task period and condition. These averaged values were analyzed using an LMEM with task period, condition, and their interaction as fixed effects, and channel pair nested within participants as random effects:

$$ {\text{MeanNetGranger }}\sim { 1 } + {\text{ taskPeriod }}*{\text{ condition }} + \, \left( {{1 }|{\text{ patient}}} \right) \, + \, \left( {{1 }|{\text{ patient}}:{\text{channelPair}}} \right) $$

## Results

### Task Performance

All 9 patients completed the task (80 trials for the same condition and 80 trials for the different condition; the training session was not included in the analysis). There was no significant difference in reaction time and accuracy of joystick responses between the same and different conditions (Wilcoxon signed-rank test, *P =* 0.4 for reaction time and *P =* 0.53 for accuracy). The mean ± SEM reaction time and accuracy for the same condition were 813 ± 47 ms and 98.1% ± 0.7%, respectively. For the different condition, the mean ± SEM reaction time and accuracy were 801 ± 49 ms and 97.6% ± 1%, and the mean ± SEM accuracy for the object’s identity question was 91% ± 4.3%.

### Increased Alpha Activity During the Delay in the Dorsal and Ventral Streams

**Fig. 2:**
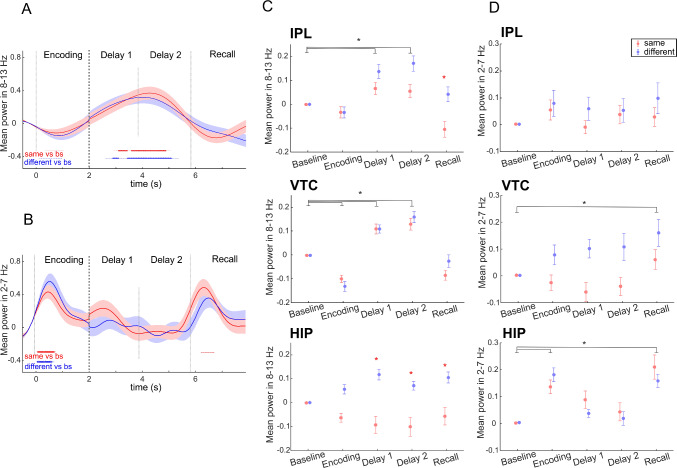
Time-frequency analysis. **A** Example of an active channel in the VTC in the alpha band (8–13 Hz). The mean ± SEM of power as a percentage of baseline activity. Red, mean responses to the "same" condition; blue, mean responses to the "different" condition. Vertical lines mark the boundaries of the task periods; the last 3.9 s of delay are shown, without the 0.0–0.2s jitter at the beginning. *Significant time points as compared to the baseline period (red, the same condition; blue, the different condition) using the FDR-corrected Wilcoxon rank sum test at *P <*0.05. **B** Example of the active channel in the HIP in the theta band (2–7 Hz). Same conventions as in panel A. **C** Mean ± SEM across channels of alpha power (8–13 Hz) for each task period in three brain regions: IPL (44 channels), VTC (64 channels), and HIP (20 channels). Red, the "same" condition; blue, the "different" condition. *Black, significant differences between task periods; red, significant differences between conditions within the same task period (*P <*0.008, LMEM). **D** Mean ± SEM across channels of theta power (2–7 Hz) for each task period in three brain regions: IPL (31 channels), VTC (29 channels), and HIP (18 channels). The same conventions as in panel C. Alpha power increases during the delay in both dorsal and ventral streams, while theta power increases only in the ventral stream.

Based on visual analysis of time-frequency spectrograms (Fig. 1C), prominent changes during the delay were found in the alpha band. Mean alpha power at 8–13 Hz was extracted for the whole length of the epoch (−500 ms to 7800 ms) for the same and different conditions and compared with the baseline (500 ms of pre-trial fixation period) to find active channels in this frequency band (an exemplary channel is shown in Fig. 2A). Then, the mean alpha power across each task period and both conditions from all active channels in the three brain regions (a total of 128 channels, see Table 1) from 9 patients was tested for significant differences by the LMEM (one model for each brain region).

The LMEM showed (Fig. 2C, see Table S2 for further details) that in the IPL and VTC, alpha power increased for both conditions during the two parts of the delay compared to the baseline (*IPL*: main effect of task period Delay 1 *t*(430) = 4.16, *P <*0.001, task period Delay 2 *t*(430) = 5.18, *P <*0.001; *VTC*: task period Delay 1 *t*(630) = 4.46, *P <*0.001, task period Delay 2 *t*(630) = 6.48, *P <*0.001). In the IPL, there was also a difference between the same and different conditions, with the same condition showing a decrease in alpha power during recall (interaction between task period Recall and condition *t*(430) = 3.14, *P* = 0.002). In the HIP, alpha power increased only for the different condition during the two parts of the delay and recall (interaction between task period Delay 1 and condition *t*(190) = −4.57, *P <*0.001, interaction between task period Delay 2 and condition *t*(190) = −3.73, *P <*0.001, interaction between task period Recall and condition t(190) = −3.51, *P <*0.001).

### Increased Theta Power in the Ventral Stream

We also extracted mean theta power at 2–7 Hz for the entire length of the epoch. Similar to the alpha power analysis, we identified active channels in this frequency band (an exemplary channel is shown in Fig. 2B). We averaged the 2–7 Hz power across each task period and each condition from all active channels in the three brain regions (a total of 78 channels, see Table 1) from 9 patients and tested the effect of task period and condition for significant differences using the LMEM.

The LMEM showed that, in the IPL, there was no significant difference in 2–7 Hz power between conditions and task periods or their interactions (Fig. 2D, see Table S3 for further details). In the VTC, theta power increased for both conditions during recall compared to the baseline (the main effect of task period Recall *t*(280) = 3.51, *P <*0.001). During the delay periods, theta power in the VTC showed a tendency to increase only in the different condition, although this did not reach statistical significance after Bonferroni correction (interaction between task period Delay 1 and condition *t*(280) = −2.54, *P* = 0.012; interaction between task period Delay 2 and condition *t*(280) = −2.31, *P* = 0.022). In the HIP, theta power also increased for both conditions during the recall, but also during the encoding (main effect of task period Recall *t*(170) = 4.91, *P <*0.001; main effect of task period Encoding *t*(170) = 5.63, *P <*0.001). In the VTC and HIP, the main effect of the condition and the interaction between each task period and condition were not significant.

### Functional Coupling Between the Dorsal and Ventral Streams

**Fig. 3:**
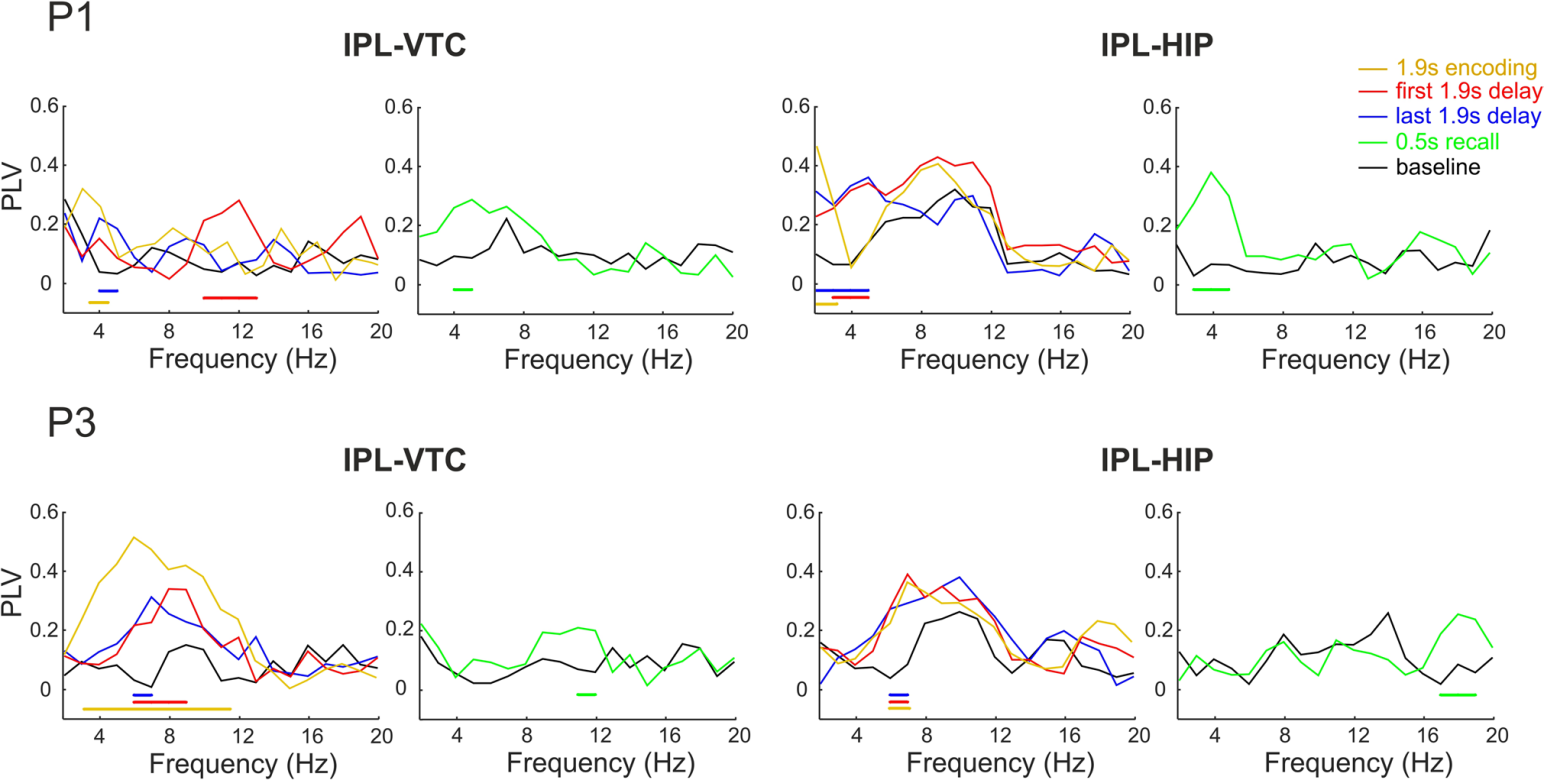
PLV spectra for individual channel pairs between IPL and VTC, and between IPL and HIP during baseline (black), encoding (yellow), first half of the delay (red), second half of the delay (blue), and 0.5 s of the recall phase (green) (examples for patients P1 and P3). Horizontal colored bars indicate frequency bins of significant PLV differences from baseline (*P <*0.05, cluster-based non-parametric permutation test): yellow for encoding, red for the first half of the delay, blue for the second half, and green for the recall. Different channel pairs exhibit significant PLV differences from baseline at various frequencies within the 2–20 Hz range.

To investigate whether the dorsal and ventral streams communicate and when during memory-guided actions, we first calculated the PLV across all trials for each channel pair between the IPL and VTC, and between the IPL and HIP within the same hemisphere for all task periods. The numbers of channels and patients in each brain region included in the PLV analysis are shown in Table 2. To determine the statistical significance of PLV at each frequency bin, the PLV spectra for each period were compared to the baseline. We found that different channel pairs exhibited significant PLV at various frequency points within the 2–20 Hz range (Fig. 3).

**Table 2:** Information on patients and channels used in the PLV analysis

Patient	IPL-VTC, hemisphere	Channels (IPL/VTC)	IPL-HIP, hemisphere	Channels (IPL/HIP)
P1	R	19/18	R	19/5
P2	–	–	–	–
P3	R	13/19	R	13/3
P4	–	–	R	4/11
P5	L	6/34	R	7/9
P6	L	7/15	L	7/8
P7	R	33/4	–	–
P8	R	6/16	–	–
P9	L	21/44	L	21/5

**Fig. 4:**
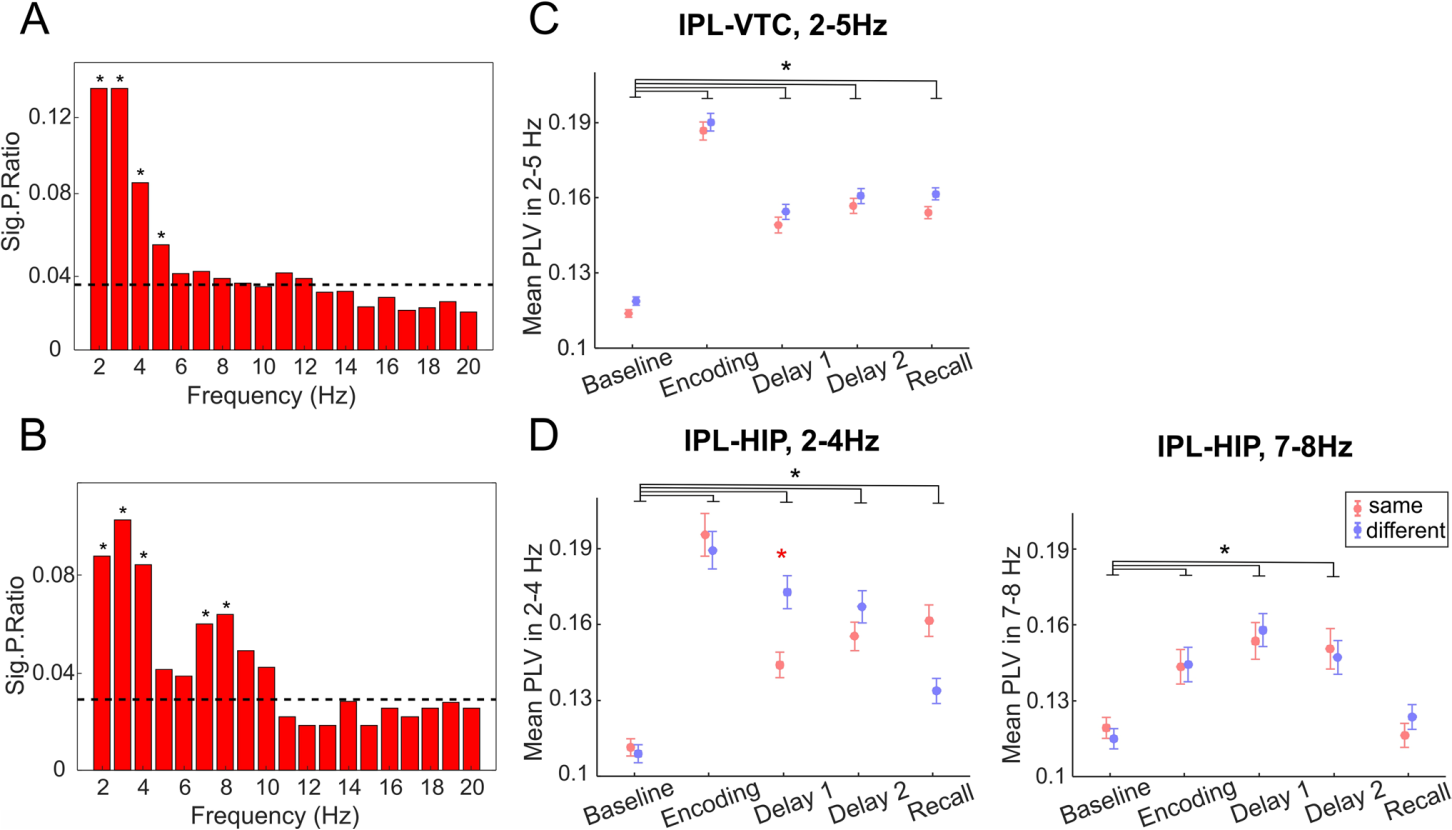
PLV analysis.** A** Sig.P.Ratio between IPL and VTC across frequency bins (1-Hz intervals). The Sig.P.Ratio represents the proportion of channel pairs across all patients exhibiting significant PLV in each frequency bin. Dashed black line, the median Sig.P.Ratio across all frequency bins. *Frequency bins with a significantly higher Sig.P.Ratio than the median (binomial test, *P <*0.05). **B** Sig.P.Ratio between IPL and HIP across frequency bins (1-Hz intervals). The legend and description are consistent with panel A. **C** Means and SEMs of PLV in the 2–5 Hz range for each task period between IPL and VTC (755 channel pairs). Red, the "same" condition; blue, the "different" condition. *Black, significant differences between task periods; red, significant differences between conditions within the same task period (*P <*0.0167, LMEM). **D** Means and SEMs of PLV in the 2–4 Hz and 7–8 Hz ranges for each task period between IPL and HIP (134 channel pairs). The legend and description are consistent with panel C. Significant increases in PLV occurred between IPL and VTC in the 2–5 Hz range and between IPL and HIP in the 2–4 Hz and 7–8 Hz ranges during encoding and delay, suggesting functional coupling between these regions. Notably, IPL-HIP connections showed a higher PLV in the 2–4 Hz range for the "different" condition during the first half of the delay.

Next, for each pair of brain regions, we identified the frequency ranges in which most channel pairs showed significant differences relative to the baseline. To this end, we compared the ratios of significant pairs (Sig.P.Ratios) between the regions at each frequency point with the median Sig.P.Ratio across all frequency points in the region pair using binomial tests. For IPL-VTC connections, the above-chance proportions of significant channel pairs were in the 2–5 Hz range (*P <*0.05, FDR-corrected, Fig. 4A). For IPL-HIP connections, above-chance proportions of significant channel pairs were identified in the 2–4 Hz and 7–8 Hz ranges (Fig. 4B).

### Functional Coupling Between IPL and VTC

For all significant IPL-VTC connections (755 channel pairs in 7 patients), we calculated the PLV separately for each condition and averaged the PLV over the 2–5 Hz range for each task period (baseline, encoding, two parts of the delay, and recall). These averaged values were then analyzed using an LMEM. The LMEM showed (Fig. 4C, see Table S4 for details) that PLV increased for both conditions during the encoding, the two parts of the delay, and recall compared to the baseline (main effect of task period Encoding t(6780) = 23.06, *P <*0.001, task period Delay 1 *t*(6780) = 11.57, *P <*0.001, task period Delay 2 *t*(6780) = 13.61, *P <*0.001, task period Recall *t*(6780) = 13.86, *P <*0.001). A *post-hoc* test for the main effect of the task period showed that PLV was significantly higher during Encoding than during Delay 1, Delay 2, and Recall (all *P <*0.001). The main effect of the condition and the interaction between each task period and condition were not significant.

### Functional Coupling Between IPL and HIP

For all significant IPL-HIP connections (134 channel pairs in 6 patients), we calculated the PLV separately for each condition and first averaged the PLV over the 2–4 Hz range for each task period (baseline, encoding, two parts of the delay, and recall). The LMEM showed (Fig. 4D, see Table S4 for details) that PLV increased for both conditions during the encoding, the two parts of the delay, and recall compared to the baseline (main effect of task period Encoding *t*(1330) = 10.76, *P <*0.001, main effect of task period Delay 1 *t*(1330) = 8.54, *P <*0.001, task period Delay 2 *t*(1330) = 7.78, *P <*0.001, task period Recall *t*(1330) = 3.32, *P <*0.001). Furthermore, there was a significant difference between the same and different object conditions during the first half of the delay and on the verge of significance during the recall. During the first half of the delay, PLV for the different conditions was higher, while during the recall, PLV for the same condition was higher (interaction between task period Delay 1 and condition *t*(1330) = −2.97, *P* = 0.003, interaction between task period Recall and condition *t*(1330) = 2.39, *P* = 0.0169).

Next, we analyzed the IPL-HIP connections in the 7–8 Hz range, which also had an above-chance proportion of significant channel pairs. The LMEM for this frequency range showed that the main effect of condition and the interaction between each task period and condition were not significant (Fig. 4D). However, for both conditions, PLV increased during the encoding and the two parts of the delay compared to the baseline (main effect of task period Encoding *t*(1330) = 4.13, *P <*0.001, main effect of task period Delay 1 *t*(1330) = 6.04, *P <*0.001, task period Delay 2 *t*(1330) = 4.53, *P <*0.001.

### Directed Functional Coupling Between the Dorsal and Ventral Streams

To further investigate the directionality of information flow during the encoding and delay phases, we used spectral GC analysis as a measure of directed functional connectivity between the VTC and IPL, and between the IPL and HIP. We focused our analysis on the same channel pairs that showed significant PLV in the previous analysis, thus concentrating on the most relevant connections and also allowing their direct comparison.

**Fig. 5:**
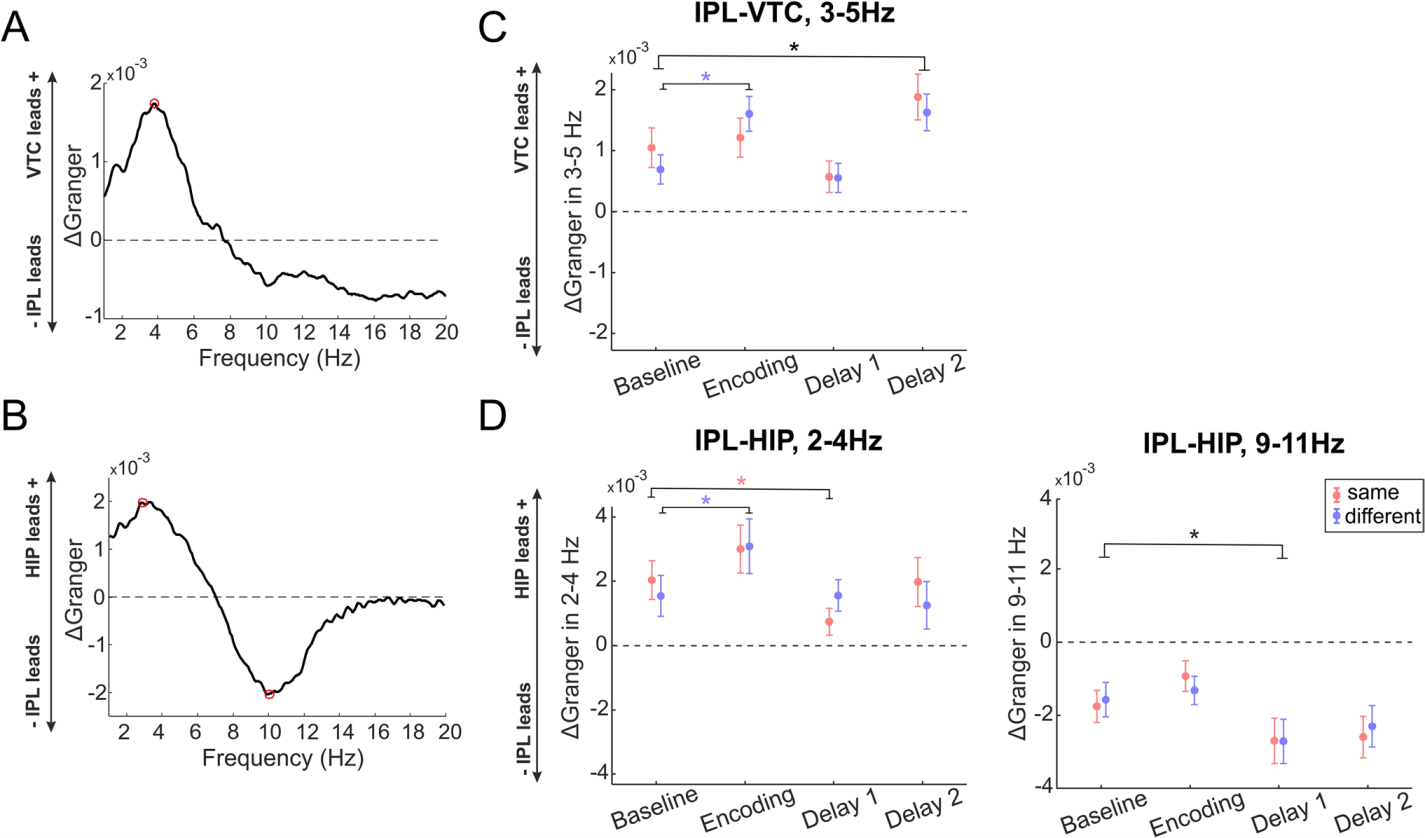
Spectral Granger causality analysis. **A** Mean net GC difference (ΔGranger) between VTC and IPL connections across 1–20 Hz. ΔGranger is calculated as GC_VTC→IPL_ minus GC_IPL→VTC_. Positive ΔGranger values indicate a net information flow from VTC to IPL (VTC leads IPL), while negative values indicate a net flow from IPL to VTC. Dashed horizontal line, ΔGranger = 0 (no net directionality); red circle, the frequency at which the maximal net information flow between VTC and IPL occurs. **B** Mean net ΔGranger between HIP and IPL connections across 1–20 Hz. The conventions are the same as in panel A. Positive values indicate HIP leads IPL; negative values indicate IPL leads HIP. **C** Mean (± SEM) net ΔGranger in the 3–5 Hz frequency range between VTC and IPL for four task periods—baseline, encoding, delay 1, and delay 2—in both "same" (red) and "different" (blue) conditions (755 channel pairs). *Black, significant differences between task periods that are consistent across both conditions (*P <*0.05, LMEM); blue, significant differences between task periods only in the "different" condition; red significant differences only in the "same" condition. **D** Mean (± SEM) net ΔGranger in the 2–4 Hz and 9–11 Hz frequency range between HIP and IPL for the four task periods in both "same" (red) and "different" (blue) conditions (134 channel pairs). The legend and conventions are the same as in panel C. Significant differences were determined using linear mixed-effects models and *post-hoc* tests (*P <*0.05). The VTC exerts a greater influence on the IPL in both conditions during the second half of the delay and especially during encoding in the "different objects" condition. The HIP tends to lead the IPL during encoding in the slow theta range, while IPL influences HIP during the delay in the alpha range, highlighting dynamic and frequency-specific directional interactions that vary across task phases.

First, we identified the frequencies at which the net information flow between the two directions was maximal. For the VTC-IPL connections, the peak net GC occurred at ~4 Hz (Fig. 5A), and we focused on the frequency range of 3–5 Hz in subsequent analyses of these connections. Interestingly, for the HIP-IPL connections, we found two peaks of maximal net GC in opposite directions: one at ~3 Hz and another at ~10 Hz (Fig. 5B). Consequently, we concentrated on the frequency ranges of 2–4 Hz and 9–11 Hz for the HIP-IPL connections in further analyses.

### Directed Functional Coupling Between IPL and VTC

We averaged the net GC values over the 3–5 Hz range for each task period—baseline, encoding, delay 1, and delay 2—and for each condition (same and different objects) (see also supplementary Fig. S2 for the GC for each direction separately). These averaged values were then analyzed using an LMEM. The LMEM results (Fig. 5C; see Table S5 for details) revealed that during the second half of the delay (Delay 2), the mean net GC was significantly higher than the baseline for both conditions (main effect of task period Delay 2: *t*(6032) = 2.91, *P* = 0.0037). This suggests that during Delay 2, the VTC exerted a greater Granger-causal influence on the IPL than in the baseline period, likely providing the IPL with visual information needed for the upcoming action.

In addition, during the encoding phase, there was a trend suggesting that the VTC had a larger GC influence on the IPL in the condition of the different objects compared to the same object condition. The interaction between the encoding period and condition was on the verge of statistical significance (*t*(6032) = 1.84, *P* = 0.0655). *Post-hoc* tests showed that, for the different object conditions, the net GC during encoding was significantly higher than during the baseline (*P* = 0.0015). This suggests that when participants had to remember both object identity and location, the VTC's influence on the IPL was greater during encoding.

It is also noteworthy that the intercept of the LMEM was significant [*t*(6032) = 2.84, *P* = 0.0045], indicating that even during the baseline period, there was a net GC influence from the VTC to the IPL for both conditions. This baseline influence may reflect ongoing processing or inherent connectivity between these regions independent of the task.

### Directed Functional Coupling Between IPL and HIP

Similarly, we analyzed the net GC between the IPL and HIP and averaged values over the 2–4 Hz and 9–11 Hz ranges for each task period and condition, and these averaged values were then analyzed using an LMEM (see also Fig. S2 for the GC for each direction separately).

In the 2–4 Hz frequency range, the LMEM results (Fig. 5D; see Table S5 for details) indicated that during Delay 1, the mean net GC was slightly lower than the baseline in the same objects condition [*t*(1064) = −2.13, *P* = 0.0331]. In the different objects condition, there was no significant change in net GC during Delay 1 compared to baseline (*post-hoc* test *P* = 0.9834). This suggests that during Delay 1, the net GC between the HIP and IPL remained similar to baseline levels or was slightly reduced at 2–4 Hz. During the encoding phase, in the different condition, the mean net GC was significantly higher than during the baseline (*post-hoc* test: *P* = 0.0109) and there was a positive estimate indicating a trend toward increased net GC also for the same condition [*t*(1064) = 1.60, *P* = 0.1101], though this did not reach statistical significance (see also supplementary Fig. S2C). These findings suggest that during encoding at 2–4 Hz, the hippocampus tends to lead the IPL in both conditions, with a stronger effect in the 'different' condition.

In the 9–11 Hz frequency range, the LMEM revealed that during Delay 1, the mean net GC was significantly lower than the baseline for the same [*t*(1064) = −2.17, *P* = 0.0301] and different condition (post-hoc test *P* = 0.0086). This indicates an increased influence from the IPL to the HIP during Delay 1. Also, during Delay 2, there was a trend toward decreased net GC, approaching significance [*t*(1064) = −1.93, *P* = 0.0534], suggesting that the IPL may continue to influence the hippocampus during this period, though this effect was not statistically significant (see also Fig. S2D).

These findings suggest the frequency-specific and task-dependent directional interactions between the hippocampus and IPL. During encoding, the hippocampus tends to lead the IPL in both conditions in the 2–4 Hz range, but especially when object identity information is involved. During memory maintenance, especially in the initial phase, the IPL leads the hippocampus in both conditions in the 9–11 Hz range, which may be crucial for maintaining memory representations over the delay.

## Discussion

Our study aimed to investigate the neural dynamics and functional connectivity between the dorsal and ventral streams, as well as the hippocampus, during memory-guided actions involving object identity and location information. Using iEEG recordings from nine patients performing a delayed action task, we tested four hypotheses related to the roles and interactions of these brain regions. First, as we hypothesized, we found sustained activity in both the dorsal (IPL) and ventral (VTC) streams throughout the delay phase, reflected by increased alpha power (8–13 Hz) for both the same and different object conditions. In addition, there was a tendency for theta power (2–7 Hz) to increase in the VTC during the delay period, but only in the "different objects" condition. Second, we also hypothesized that increased synchronization between IPL and VTC would indicate integrated processing of spatial information. This was supported by the increased PLV between IPL and VTC in the slow theta range throughout the encoding, delay, and recall periods for both conditions. Third, we predicted additional activity in the hippocampus and stronger IPL-hippocampus synchronization during the delay in the "different objects" condition, which involves remembering both object location and identity information. Our prediction was supported by an increase in alpha power in the hippocampus only for the different object conditions, and by increased PLV between IPL and hippocampus in the slow and fast theta ranges, with a notable difference between the same and different conditions in the slow theta. Finally, we hypothesized that the directionality of interactions between these regions would vary across task periods. GC analysis revealed that during Delay 2, the VTC exerted a greater influence on the IPL in both conditions and during the encoding, particularly in the different object conditions. In addition, the hippocampus tended to lead the IPL during encoding in the slow theta, while the IPL influenced the hippocampus during the delay in the alpha range.

Our finding of increased alpha power in both the IPL and VTC throughout the delay reflects the involvement of both streams in working memory maintenance for memory-guided actions, and supports the new framework of their combined role in processing visual information for delayed actions [13]. Previous studies using perceptual spatial judgments without a delay showed dorsal stream activation in the superior and inferior parietal lobules ([39–41], see also review [25]). In contrast, our task, which involves memory-guided actions, activated both the dorsal and ventral streams. These areas most likely maintain spatial information, i.e., the location of the object that is closer to the central cross, as suggested by no differences between the same and different object conditions in the alpha range during the delay period in IPL and VTC. The ventral stream activation may also be related to the conversion of transient egocentric into stable allocentric representations. Distance estimation in our task was likely performed using egocentric coordinates, as the cross was always centered on the screen and served as a stable reference point, aligning with the participant’s midsagittal plane. Both the original and revised models link the dorsal and ventral streams with egocentric and allocentric representations, respectively. Egocentric representations, associated with the dorsal stream, are transient and support real-time actions, while allocentric representations, linked to the ventral stream, are long-lasting and serve as a basis for memory-guided actions [2, 42]. This shift is supported by behavioral studies showing better performance in allocentric than egocentric frames after brief delays [43, 44]. Moreover, a patient with occipito-parietal damage showed deficits only in the egocentric condition after a 1.5-s delay, but not after 5 s [45], suggesting that over time, egocentric may transform into allocentric representations, activating the ventral stream.

The increased alpha activity during the delay and the increase in theta power during encoding and recall highlights the dynamic role of brain oscillations in memory processes. The increase in alpha amplitude aligns with other studies showing similar increases during working memory tasks [8–10, 17]. Although alpha oscillations have often been interpreted as reflecting cortical idling [46], recent studies suggest that increased alpha amplitude may be associated with active inhibition of task-irrelevant information and attentional processes [47–50]. Increased alpha power with memory load in working memory tasks further challenges the idling hypothesis and may suggest that alpha band oscillations are directly involved in memory maintenance [48, 51]. In line with these studies, the increase in alpha power in our task likely reflects either memory maintenance or active inhibition of irrelevant information, enhancing the processing of relevant spatial information in the dorsal and ventral streams. The increase in theta power in the VTC during the delay periods in the "different objects" condition suggests that theta-band activity in the ventral stream may support the maintenance of object identity information, particularly when both position and identity need to be remembered. This is consistent with recent findings [52] showing that during the maintenance of visual working memory, the ventral visual stream matches representations in lower frequency bands, likely storing detailed visual information when it is about to be used. On the other hand, the increase in theta power during encoding and recall for both conditions, particularly in the hippocampus, is likely associated with memory formation about the objects and their spatial relations and successful retrieval [53–56].

To investigate whether the recorded neural dynamics were specific to memory-guided actions, we analyzed the immediate trials, where participants reached for the object without any delay (see Supplementary Results). In contrast to delayed trials, immediate trials showed decreases in alpha and theta power in the IPL and VTC, with no significant changes in the hippocampus. This suggests that the increase in alpha and theta power during delayed trials is associated with memory maintenance and that the hippocampus is primarily engaged when working memory processes are involved. The decreased alpha and theta power in the IPL and VTC likely reflects cortical activation associated with attention and visuomotor processing required for immediate action [50, 57, 58]. Alpha desynchronization has been associated with increased attentional demands and sensory processing during tasks requiring the processing of external stimuli [57, 59]. Notably, the decrease in alpha and theta power in the VTC was greater in the "different objects" condition than in the "same objects" condition, suggesting that the VTC is more engaged when discriminating between different objects. Given the role of the VTC in the processing of complex visual stimuli and object recognition [60, 61], this greater decrease indicates higher visual processing demands and selective attention for object identity.

Next, as we hypothesized, we found a significant increase in PLV between IPL and VTC during the delay, but also during the encoding and recall period in the slow theta (2–5 Hz) range. Previous research has shown that oscillations in this range facilitate the coordination of distant brain regions for cognitive functions, including working memory and attention [17, 19–22]. The highest PLV between the IPL and VTC was recorded during encoding, indicating long-range communication that supports the integration of spatial object information essential for forming memory representations. Although there were no significant differences in PLV between the "same objects" and "different objects" conditions, suggesting that this functional coupling is primarily driven by spatial processing common to both, GC analysis showed that during encoding, the VTC tended to have a stronger influence on the IPL in the "different" condition. This pattern suggests that when participants need to remember both object identity and location, the influence of the ventral stream on the dorsal stream increases with the processing of more complex visual stimuli [61]. Furthermore, the VTC exerted a greater influence on the IPL in both conditions during Delay 2, suggesting that the ventral stream provides critical visual information to the dorsal stream in preparation for the upcoming action. These findings align with our hypotheses and support an integrated model of dorsal and ventral stream interaction [13, 62], highlighting dynamic and directional communication between the VTC and IPL during memory-guided actions.

Our data support the hypothesis that the hippocampus is involved in processing and maintaining information about an object’s identity and position during memory-guided actions. This is supported by our finding of an increase in alpha power in the hippocampus only for the different object conditions. Although the reaction time and accuracy of reaching responses did not differ between the same and different object conditions, the different conditions were more challenging, requiring the memory of both the position and identity of the object. This aligns with studies on working memory capacity, showing that higher task demands lead to stronger medial temporal lobe involvement, particularly in the hippocampus [17, 63, 64]. Hippocampal maintenance neurons have been shown to increase firing rates with higher workloads in both verbal and visual working memory tasks [17, 64], and although we did not record from single neurons, the population-level alpha power increase throughout delay aligns with findings linking increases in alpha power magnitude with memory load [48, 51]. Notably, such alpha activity in the hippocampus was absent in the immediate trials, where there was no need to hold the memory of the position and identity of two related objects over several seconds.

This hypothesis is further supported by our results of connectivity analysis. The increased PLV between the IPL and hippocampus was recorded in both slow theta (2–4 Hz) and fast theta (7–8 Hz) frequencies during encoding and both parts of the delay. During Delay 1, synchronization in the slow theta range was higher for the different conditions. This difference in PLV suggests condition-dependent functional connectivity, with the hippocampus potentially modulating the processing of spatial and object identity information at the beginning of the delay phase, particularly in slow theta. This aligns with other studies highlighting the role of the hippocampus in supporting memory for multiple types of interrelated information [16, 65]. In contrast, no difference between conditions was found in the PLV in the fast theta range (7–8 Hz). The distinct roles of slow and fast theta oscillations in our task highlight the functional differentiation within the theta band. Slow theta oscillations may be more involved in integrating and processing specific task-related information, while fast theta oscillations might support broader cognitive functions required for sustained task engagement. This distinction is supported by several studies showing different characteristics of slow and fast theta [66, 67]. For instance, an iEEG study showed that slow theta in the human hippocampus has higher power during successful memory encoding and is functionally associated with gamma oscillations, but this result was not found for fast theta [66]. Also, slow theta oscillations in the human hippocampus appear to be similar to the memory-related theta oscillations recorded in animals [68].

The GC results complement these findings and suggest dynamic, frequency-specific interactions between the hippocampus and IPL. During encoding, the hippocampus tends to lead the IPL in the slow theta (2–4 Hz) range, especially when object identity information is involved, facilitating the exchange of object identity and spatial information through hippocampal-dorsal stream interactions. During memory maintenance, the IPL leads the hippocampus in the alpha (9–11 Hz) range, suggesting that the dorsal stream supports the active maintenance and top-down control of memory contents [47].

Despite its strengths, our study has several limitations. The sample size of nine epilepsy patients, while typical for iEEG studies [9, 37, 69], may limit the generalizability of the findings to a broader population. A significant limitation of iEEG studies is the variation in implantation schemes across patients. In this study, more electrodes were implanted in the right hemisphere, and different hemispheres were used in PLV analysis among patients, potentially influencing the results. In addition, due to temporal smoothness, low-frequency oscillations are inherently more likely to produce higher PLV values than faster oscillations. To mitigate this potential bias, we focused on the differences between task and baseline PLV and applied permutation testing with cluster correction [22, 24]. The limited number of channels covering the hippocampus and IPL simultaneously may have reduced the statistical power to detect significant effects, particularly in the GC analysis of IPL–hippocampus interactions. However, in our study, each conclusion is based on data from at least six patients, providing a solid foundation for our results, given the high signal-to-noise ratio of iEEG recordings and the fact that reproducible results from the same brain structures across a few subjects are sufficient for scientific reports [70]. Future studies with larger sample sizes and more extensive electrode coverage could provide more definitive conclusions regarding directional interactions between the hippocampus and the IPL. Another consideration is the block design, which separates the "same" and "different" conditions into distinct blocks. While this ensured clarity in instructions and task design, it may have introduced systematic biases, such as participants adopting different strategies for each block. A mixed design with randomized conditions could address this limitation in future studies.

In conclusion, our study provides electrophysiological evidence supporting a more integrated role of the dorsal and ventral streams in memory-guided actions, with both streams contributing to the maintenance and processing of spatial information during working memory. Furthermore, the hippocampus seems to be involved when not only spatial but also identity information needs to be maintained. The dynamic and frequency-specific directional interactions among the IPL, VTC, and hippocampus, as demonstrated by our PLV and GC analyses, highlight the complexity of neural communication underlying memory-guided actions. These findings challenge the traditional dichotomy of the perception-action model and support a new framework in which both streams contribute to memory-guided actions, and that their interactions are particularly important [13]. Understanding these interactions may have potential clinical implications for developing interventions aimed at enhancing visuomotor coordination and memory functions in patients with neurological disorders affecting these brain regions.

## Supplementary Information

Below is the link to the electronic supplementary material.

Supplementary file1 (PDF 542 KB)

## Data Availability

The raw iEEG data that support the findings of this study are available in the Zenodo repository https://zenodo.org/records/13712628. Further data are available upon reasonable request from the corresponding author. The codes used to produce the results in the paper are freely available at the repository https://github.com/kamilvlcek/iEEG_scripts/tree/v3.1.0.
