# Correction: Neural Dynamics of Visual Stream Interactions During Memory-Guided Actions Investigated by Intracranial EEG

**DOI:** 10.1007/s12264-025-01453-w

**Published:** 2025-07-16

**Authors:** Sofiia Moraresku, Jiri Hammer, Vasileios Dimakopoulos, Michaela Kajsova, Radek Janca, Petr Jezdik, Adam Kalina, Petr Marusic, Kamil Vlcek

**Affiliations:** 1 Laboratory of Neurophysiology of Memory, Institute of Physiology, Czech Academy of Sciences, Prague, Czechia; 2 Third Faculty of Medicine, Charles University, Prague, Czechia; 3 Department of Neurology, Second Faculty of Medicine, Charles University and Motol University Hospital, Member of the Epilepsy Research Centre Prague – EpiReC Consortium, Prague, Czechia; 4 Department of Circuit Theory, Faculty of Electrical Engineering, Czech Technical University in Prague, Member of the Epilepsy Research Centre Prague – EpiReC Consortium, Prague, Czechia; 5 Klinik fur Neurochirurgie, Universitatsspital Zurich, Universitat Zurich, Zurich, Switzerland

**Correction to: Neuroscience Bulletin** 10.1007/s12264-025-01371-x

In this article the affiliation “Department of Circuit Theory, Faculty of Electrical Engineering, Czech Technical University in Prague, Member of the Epilepsy Research Centre Prague - EpiReC Consortium, Prague, Czechia” should only be assigned to Radek Janca and Petr Jezdik.

It is removed from the authors: Jiri Hammer, Michaela Kajsova, Adam Kalina, Petr Marusic, and Kamil Vlcek.

Sofiia Moraresku^1,2^, Jiri Hammer^3,4^, Vasileios Dimakopoulos^5^, Michaela Kajsova^3,4^, Radek Janca^4^, Petr Jezdik^4^, Adam Kalina^3,4^, Petr Marusic^3,4^, Kamil Vlcek^1,3,4^.

Should read as

Sofiia Moraresku^1,2^, Jiri Hammer^3^, Vasileios Dimakopoulos^5^, Michaela Kajsova^3^, Radek Janca^4^, Petr Jezdik^4^, Adam Kalina^3^, Petr Marusic^3^, Kamil Vlcek^1,3^.

