# Supplementary material for: Neural Dynamics of Visual Stream Interactions During Memory-Guided Actions Investigated by Intracranial EEG
Source: Neurosci Bull. 2025 Mar 17;41(8):1347–63. doi: 10.1007/s12264-025-01371-x (PMC12314303; doi:10.1007/s12264-025-01371-x)
Supplement: Supplementary file 1 — Supplementary file1 (PDF 542 KB) [file 12264_2025_1371_MOESM1_ESM.pdf]

**Supplementary Material**

**Supplementary Results**

**Time-frequency analysis of the immediate trials**

In addition to the delayed trials, our experiment included 160 immediate trials, equally divided between the 'same' and 'different' conditions, in which participants reached for the object immediately without any delay. As a control analysis, we applied time-frequency analysis to these trials to determine whether the neural dynamics recorded in the delayed trials were specifically associated with the delay period.

In the immediate trials, encoding and action execution occurred simultaneously within a trial duration of 2 s, unlike the delayed trials where these processes were temporally separated by a delay period. Due to this overlap, direct comparisons of specific task phases between immediate and delayed trials are challenging. However, we segmented the immediate trials into two time intervals for analysis: time interval 1 (0–0.5 s) – activity associated with the stimulus presentation; and time interval 2 (0.5–1 s) – activity preceding the behavioral response, as the average reaction time of the joystick response was ~1 s after stimulus onset.

Similar to the analysis of the delayed trials, we identified active channels in the alpha (8–13 Hz) and theta (2–7 Hz) frequency bands within the VTC, IPL, and HIP. We then calculated the mean power within each band for each time interval and condition. The data were analyzed using linear mixed-effects models (LMEMs) with time interval, condition (same *vs* different), and their interaction as fixed effects, and channel and patient as random effects.

We found that in the immediate trials (Fig. S1), no significant changes in alpha and theta power were found in the hippocampus (neither the main effects of time interval and condition nor their interaction were significant in the LMEMs). In the IPL, there was a decrease in the alpha and theta power during 0.5-1 s after the stimulus compared to the baseline for both conditions (alpha power: main effect of time interval 0.5-1: $t(378) = -8.37$, $P < 0.001$; theta power: main effect of time interval 0.5-1: $t(492) = -6.18$, $P < 0.001$). In the VTC, there was a decrease in alpha power (Fig. S1A) during 0-0.5 s and 0.5-1 s (main effect of time interval 0-0.5: $t(498) = -10.87$, $P < 0.001$; time interval 0.5-1: $t(498) = -24.03$, $P < 0.001$), with a greater decrease for the different condition (interaction between time interval 0-0.5 and condition: $t(498) = 3.04$, $P = 0.002$; interaction between time interval 0.5-1 and condition: $t(498) = 7.05$, $P < 0.001$). There was also a decrease in theta power in the VTC during 0.5-1 s for both conditions (main effect of time interval 0.5-1 $t(702) = -13.86$, $P < 0.001$) and a greater decrease for the different condition during 0-0.5 s and 0.5-1 s (interaction between time interval 0-0.5

and condition $t(702) = 2.84$, $P = 0.005$; interaction between time interval 0.5-1 and condition $t(702) = 3.63$, $P < 0.001$).

To summarize, in the immediate trials, unlike the delayed trials, we found decreases rather than increases in alpha and theta power in the IPL and VTC. No significant changes were found in the hippocampus. These results suggest that the increases in alpha and theta power that occur during the delay period in the delayed trials are specifically associated with the memory maintenance required during the delay.

## Supplementary Figures and Tables

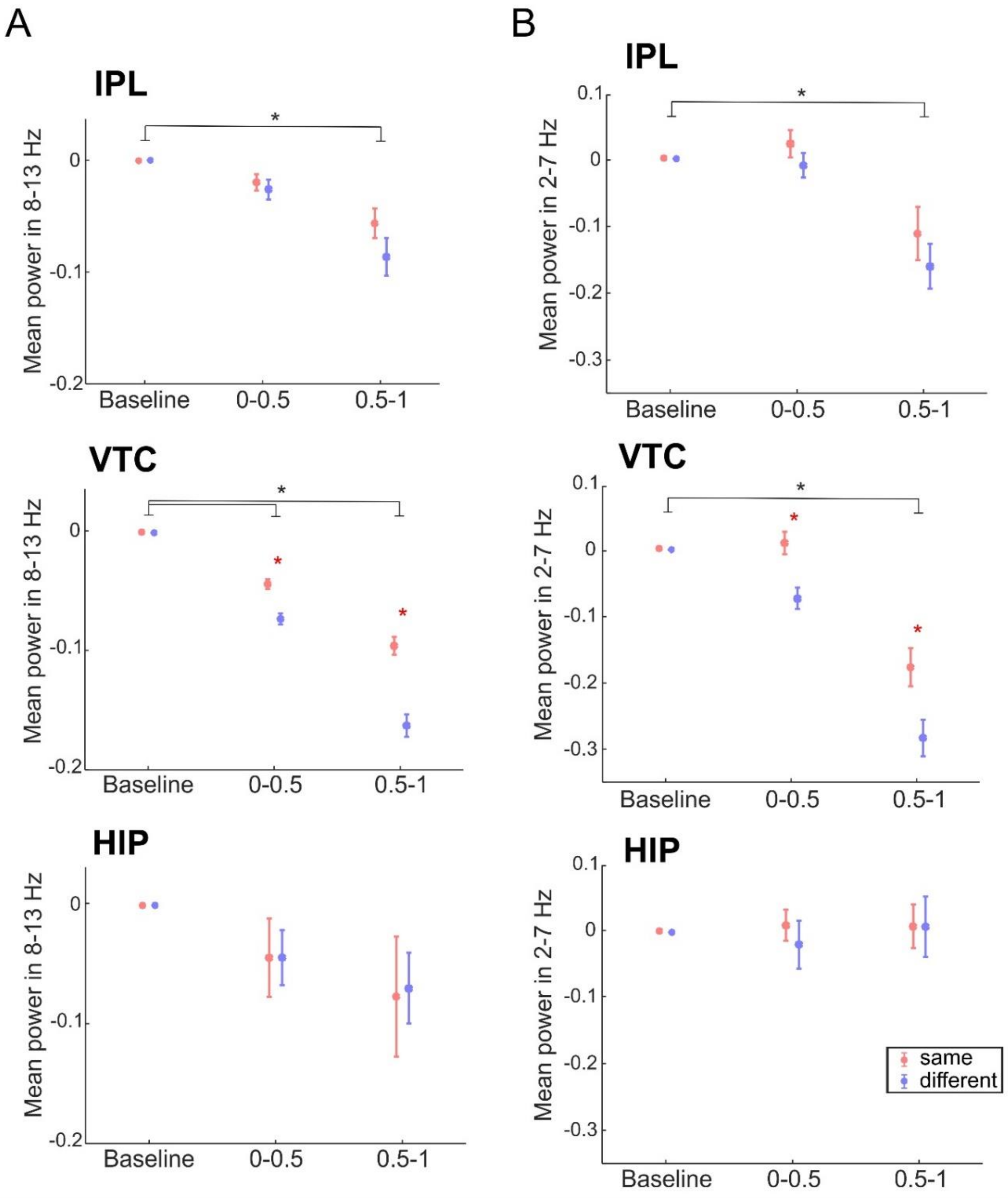

**Fig. S1. Time-frequency analysis of immediate trials. A.** Mean ± SEM of alpha power (8–13 Hz) across channels for two time intervals after stimulus onset in three brain regions: IPL (64 channels), VTC (84 channels), and HIP (6 channels). Red, the 'same' condition; blue, the 'different' condition. *Black, significant differences between time intervals and baseline; red, significant differences between conditions within the same time interval (*P* <0.008, LMEM). **B**. Mean ± SEM of theta power (2–7 Hz) across channels for two time intervals after stimulus onset in three brain regions: IPL (83 channels), VTC (118 channels), and HIP (22 channels). Same conventions as in panel A.

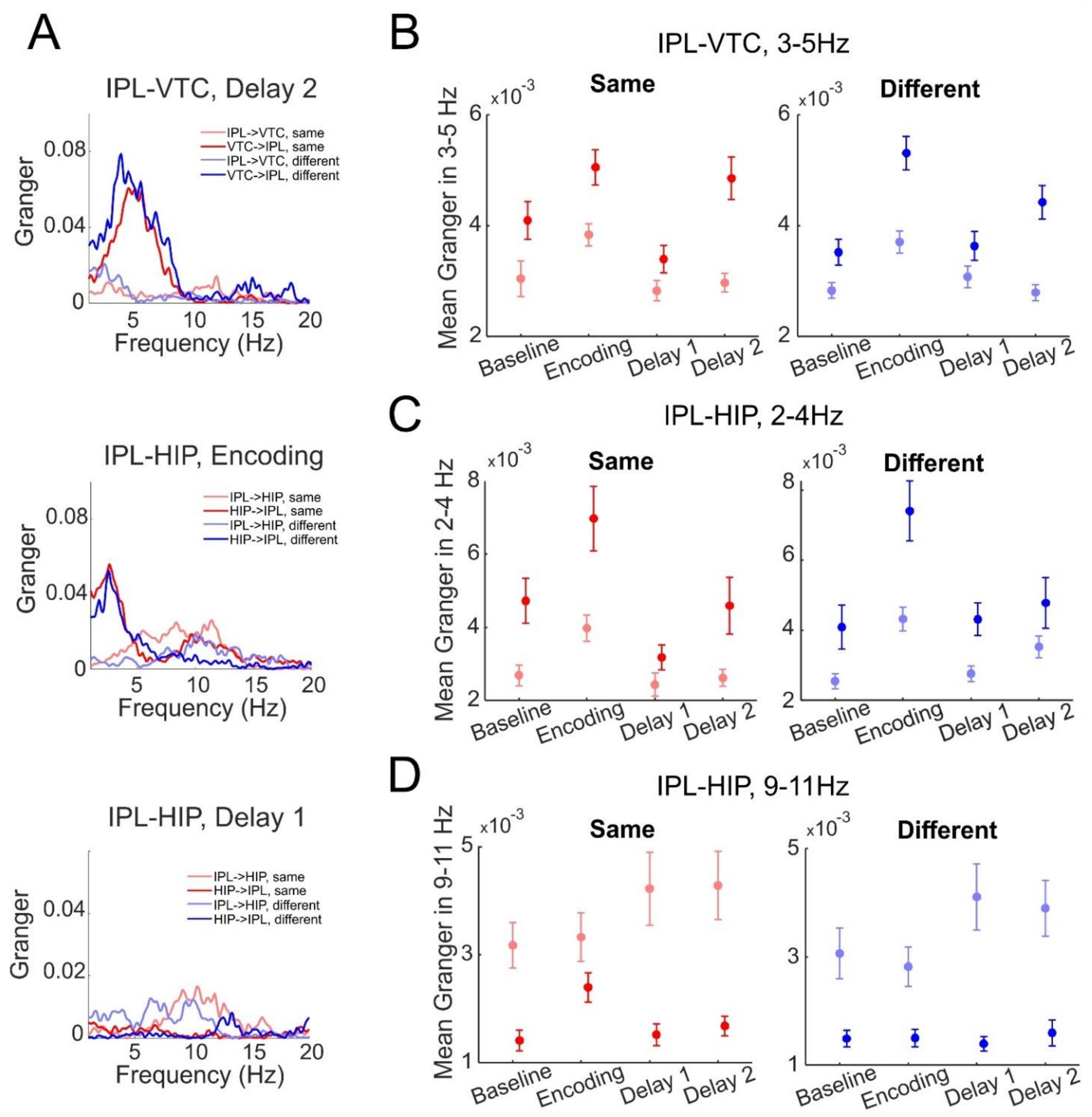

**Fig. S2. Spectral Granger Causality Analyses for IPL-VTC and IPL-HIP Connections**. **A** Exemplary Granger causality (GC) spectra for individual channel pairs in patient P3. Upper, GC between the IPL and VTC during Delay 2; middle, GC between IPL and HIP during Encoding; and lower, GC between IPL and HIP during Delay 1. Each plot illustrates the frequency-dependent GC in both directions—dark lines represent the GC from the first region to the second (e.g., VTC→IPL), and light lines represent the GC in the opposite direction (e.g., IPL→VTC). **B** Mean (± SEM) GC in the 3–5 Hz range between VTC and IPL across the four task periods—Baseline, Encoding, Delay 1, and Delay 2—for each direction and condition (755 channel pairs). In the 'same' condition, red bars represent GC from VTC to IPL, and light red bars represent GC from IPL to VTC. In the 'different' condition, blue bars represent GC from VTC to IPL, and light blue bars represent GC from IPL to VTC. **C** Mean (± SEM) GC in the 2–4 Hz range between HIP and IPL for the four task periods, displayed for each direction and condition (134 channel pairs). In the 'same' condition, red bars represent GC from HIP to IPL, and light red bars represent GC from IPL to HIP. In the 'different' condition, blue bars represent GC from HIP to IPL, and light blue bars represent GC from IPL to HIP. **D** Mean (± SEM) GC in the 9–11 Hz range between HIP and IPL for the four task periods, using the same legend and conventions as in panel C.

**Table S1. Subject information.**

| **Patient** | **Age** | **Gender** | **Handedness** | **Education** | **Epilepsy duration (years)** | **Suspected seizure zone** | **Epilepsy pathology** |
|---|---|---|---|---|---|---|---|
| P1 | 43 | M | R | primary | 33 | R temporal and parietal lobes | suspected FCD I |
| P2 | 37 | F | L | tertiary | 13 | bilateral temporal lobes | FCD I |
| P3 | 36 | M | R | tertiary | 12 | R temporal and parietal lobes, insula | post-infectious gliosis |
| P4 | 24 | F | L | primary | 8 | R temporal lobe | periventricular nodular heterotopy |
| P5 | 49 | M | R | tertiary | 27 | bilateral temporal and parietal lobes | hippocampal sclerosis |

| | | | | | | | |
|---|---|---|---|---|---|---|---|
| P6 | 30 | F | L | tertiary | 19 | L temporal lobe | periventricular nodular heterotopy |
| P7 | 46 | M | R | primary | 36 | R occipital and parietal lobes | suspected FCD I |
| P8 | 30 | M | L | primary | 28 | R operculo-insular and temporal lobe | FCD I |
| P9 | 36 | F | R | tertiary | 7 | R temporal, parietal, and occipital lobes | suspected FCD II |

F, female; M, male; R, right; L, left; FCD, Focal Cortical Dysplasia

**Table S2. Linear mixed effects model results for alpha power (8-13 Hz).**

**IPL**

| **fixed effect** | **Estimate** | **SE** | **DF** | **tStat** | **pValue** |
|---|---|---|---|---|---|
| task period Encoding | -0.034 | 0.033 | 430 | -1.03 | 0.3028 |
| task period Delay 1 | 0.137 | 0.033 | 430 | 4.16 | 0.0000 * |
| task period Delay 2 | 0.171 | 0.033 | 430 | 5.18 | 0.0000 * |
| task period Recall | 0.042 | 0.033 | 430 | 1.28 | 0.2012 |
| condition Same | 0.000 | 0.033 | 430 | -0.01 | 0.9897 |
| task period Encoding : condition Same | 0.001 | 0.047 | 430 | 0.03 | 0.9750 |
| task period Delay 1 : condition Same | -0.071 | 0.047 | 430 | -1.52 | 0.1305 |
| task period Delay 2 : condition Same | -0.116 | 0.047 | 430 | -2.47 | 0.0137 |
| task period Recall : condition Same | -0.147 | 0.047 | 430 | -3.14 | 0.0018 * |

**VTC**

| **fixed effect** | **Estimate** | **SE** | **DF** | **tStat** | **pValue** |
|---|---|---|---|---|---|
| task period Encoding | -0.130 | 0.025 | 630 | -5.23 | 0.0000 * |
| task period Delay 1 | 0.111 | 0.025 | 630 | 4.46 | 0.0000 * |
| task period Delay 2 | 0.160 | 0.025 | 630 | 6.48 | 0.0000 * |
| task period Recall | -0.024 | 0.025 | 630 | -0.97 | 0.3313 |
| condition Same | 0.000 | 0.025 | 630 | 0.01 | 0.9937 |
| task period Encoding : condition Same | 0.031 | 0.035 | 630 | 0.89 | 0.3726 |
| task period Delay 1 : condition Same | 0.000 | 0.035 | 630 | 0.01 | 0.9946 |
| task period Delay 2 : condition Same | -0.030 | 0.035 | 630 | -0.85 | 0.3930 |
| task period Recall : condition Same | -0.060 | 0.035 | 630 | -1.70 | 0.0889 |

**HIP**

| fixed effect | Estimate | SE | DF | tStat | pValue |
|---|---|---|---|---|---|
| task period Encoding | 0.056 | 0.032 | 190 | 1.72 | 0.0868 |
| task period Delay 1 | 0.117 | 0.032 | 190 | 3.63 | 0.0004 * |
| task period Delay 2 | 0.071 | 0.032 | 190 | 2.19 | 0.0295 |
| task period Recall | 0.105 | 0.032 | 190 | 3.24 | 0.0014 * |
| condition Same | -0.001 | 0.032 | 190 | -0.03 | 0.9723 |
| task period Encoding : condition Same | -0.117 | 0.046 | 190 | -2.57 | 0.0110 |
| task period Delay 1 : condition Same | -0.209 | 0.046 | 190 | -4.57 | 0.0000 * |
| task period Delay 2 : condition Same | -0.170 | 0.046 | 190 | -3.73 | 0.0003 * |
| task period Recall : condition Same | -0.160 | 0.046 | 190 | -3.51 | 0.0006 * |

* significant after Bonferroni correction, $P < 0.008$; SE, standard error; DF, degrees of freedom

**Table S3. Linear mixed effects model results for theta power (2-7 Hz).**

**IPL**

| fixed effect | Estimate | SE | DF | tStat | pValue |
|---|---|---|---|---|---|
| task period Encoding | 0.078 | 0.045 | 300 | 1.70 | 0.0894 |
| task period Delay 1 | 0.058 | 0.045 | 300 | 1.27 | 0.2064 |
| task period Delay 2 | 0.052 | 0.045 | 300 | 1.13 | 0.2581 |
| task period Recall | 0.096 | 0.045 | 300 | 2.12 | 0.0350 |
| condition Same | 0.000 | 0.045 | 300 | 0.01 | 0.9929 |
| task period Encoding : condition Same | -0.025 | 0.064 | 300 | -0.39 | 0.6979 |
| task period Delay 1 : condition Same | -0.069 | 0.064 | 300 | -1.07 | 0.2847 |
| task period Delay 2 : condition Same | -0.016 | 0.064 | 300 | -0.25 | 0.8042 |
| task period Recall : condition Same | -0.070 | 0.064 | 300 | -1.08 | 0.2790 |

**VTC**

| fixed effect | Estimate | SE | DF | tStat | pValue |
|---|---|---|---|---|---|
| task period Encoding | 0.076 | 0.045 | 280 | 1.69 | 0.0920 |
| task period Delay 1 | 0.099 | 0.045 | 280 | 2.20 | 0.0283 |
| task period Delay 2 | 0.106 | 0.045 | 280 | 2.35 | 0.0197 |
| task period Recall | 0.158 | 0.045 | 280 | 3.51 | 0.0005 * |
| condition Same | 0.001 | 0.045 | 280 | 0.01 | 0.9884 |
| task period Encoding : condition Same | -0.104 | 0.064 | 280 | -1.63 | 0.1034 |
| task period Delay 1 : condition Same | -0.162 | 0.064 | 280 | -2.54 | 0.0116 |
| task period Delay 2 : condition Same | -0.147 | 0.064 | 280 | -2.31 | 0.0218 |
| task period Recall : condition Same | -0.100 | 0.064 | 280 | -1.57 | 0.1166 |

**HIP**

| fixed effect | Estimate | SE | DF | tStat | pValue |
|---|---|---|---|---|---|
| task period Encoding | 0.177 | 0.031 | 170 | 5.63 | 0.0000 * |
| task period Delay 1 | 0.034 | 0.031 | 170 | 1.08 | 0.2825 |
| task period Delay 2 | 0.015 | 0.031 | 170 | 0.49 | 0.6269 |
| task period Recall | 0.154 | 0.031 | 170 | 4.90 | 0.0000 * |
| condition Same | -0.002 | 0.031 | 170 | -0.05 | 0.9563 |
| task period Encoding : condition Same | -0.043 | 0.045 | 170 | -0.97 | 0.3328 |
| task period Delay 1 : condition Same | 0.052 | 0.045 | 170 | 1.17 | 0.2437 |
| task period Delay 2 : condition Same | 0.026 | 0.045 | 170 | 0.58 | 0.5611 |
| task period Recall : condition Same | 0.053 | 0.045 | 170 | 1.19 | 0.2346 |

* significant after Bonferroni correction, $P<0.008$; SE, standard error; DF, degrees of freedom

**Table S4. Linear mixed effects model results for PLV.**

**IPL-VTC, 2-5 Hz**

| fixed effect | Estimate | SE | DF | tStat | pValue |
|---|---|---|---|---|---|
| task period Encoding | 0.0712 | 0.0031 | 6780 | 23.06 | 0.0000 * |
| task period Delay 1 | 0.0357 | 0.0031 | 6780 | 11.57 | 0.0000 * |
| task period Delay 2 | 0.0420 | 0.0031 | 6780 | 13.61 | 0.0000 * |
| task period Recall | 0.0428 | 0.0031 | 6780 | 13.86 | 0.0000 * |
| condition Same | -0.0049 | 0.0031 | 6780 | -1.59 | 0.1128 |
| task period Encoding : condition Same | 0.0016 | 0.0044 | 6780 | 0.36 | 0.7174 |
| task period Delay 1 : condition Same | -0.0004 | 0.0044 | 6780 | -0.10 | 0.9211 |
| task period Delay 2 : condition Same | 0.0009 | 0.0044 | 6780 | 0.21 | 0.8302 |
| task period Recall : condition Same | -0.0026 | 0.0044 | 6780 | -0.59 | 0.5544 |

**IPL-HIP, 2-4 Hz**

| fixed effect | Estimate | SE | DF | tStat | pValue |
|---|---|---|---|---|---|
| task period Encoding | 0.0802 | 0.0075 | 1330 | 10.76 | 0.0000 * |
| task period Delay 1 | 0.0637 | 0.0075 | 1330 | 8.54 | 0.0000 * |
| task period Delay 2 | 0.0580 | 0.0075 | 1330 | 7.78 | 0.0000 * |
| task period Recall | 0.0248 | 0.0075 | 1330 | 3.32 | 0.0009 * |
| condition Same | 0.0025 | 0.0075 | 1330 | 0.33 | 0.7387 |
| task period Encoding : condition Same | 0.0037 | 0.0105 | 1330 | 0.35 | 0.7247 |
| task period Delay 1 : condition Same | -0.0313 | 0.0105 | 1330 | -2.97 | 0.0031 * |
| task period Delay 2 : condition Same | -0.0141 | 0.0105 | 1330 | -1.34 | 0.1803 |
| task period Recall : condition Same | 0.0252 | 0.0105 | 1330 | 2.39 | 0.0169 |

**IPL-HIP, 7-8 Hz**

| fixed effect | Estimate | SE | DF | tStat | pValue |
|---|---|---|---|---|---|
| task period Encoding | 0.0293 | 0.0071 | 1330 | 4.13 | 0.0000 * |
| task period Delay 1 | 0.0428 | 0.0071 | 1330 | 6.04 | 0.0000 * |
| task period Delay 2 | 0.0321 | 0.0071 | 1330 | 4.53 | 0.0000 * |
| task period Recall | 0.0087 | 0.0071 | 1330 | 1.22 | 0.2216 |
| condition Same | 0.0043 | 0.0071 | 1330 | 0.61 | 0.5446 |
| task period Encoding : condition Same | -0.0052 | 0.0100 | 1330 | -0.52 | 0.6046 |
| task period Delay 1 : condition Same | -0.0086 | 0.0100 | 1330 | -0.86 | 0.3895 |
| task period Delay 2 : condition Same | -0.0009 | 0.0100 | 1330 | -0.09 | 0.9253 |
| task period Recall : condition Same | -0.0116 | 0.0100 | 1330 | -1.16 | 0.2478 |

* significant after Bonferroni correction, $P < 0.0167$; SE, standard error; DF, degrees of freedom

**Table S5. Linear mixed effects model results for net Granger.**

**IPL-VTC, 3-5 Hz**

| fixed effect | Estimate | SE | DF | tStat | pValue |
|---|---|---|---|---|---|
| intercept | 0.00142 | 0.00050 | 6032 | 2.84 | 0.0045 * |
| task period Encoding | 0.00017 | 0.00029 | 6032 | 0.58 | 0.5636 |
| task period Delay 1 | -0.00048 | 0.00029 | 6032 | -1.67 | 0.0948 |
| task period Delay 2 | 0.00083 | 0.00029 | 6032 | 2.91 | 0.0037 * |
| condition Diff | -0.00036 | 0.00029 | 6032 | -1.25 | 0.2117 |
| task period Encoding : condition Diff | 0.00075 | 0.00041 | 6032 | 1.84 | 0.0655 |
| task period Delay 1 : condition Diff | 0.00034 | 0.00041 | 6032 | 0.84 | 0.4003 |
| task period Delay 2 : condition Diff | 0.00010 | 0.00041 | 6032 | 0.26 | 0.7985 |

**IPL-HIP, 2-4 Hz**

| fixed effect | Estimate | SE | DF | tStat | pValue |
|---|---|---|---|---|---|
| intercept | 0.00195 | 0.00107 | 1064 | 1.83 | 0.0674 |
| task period Encoding | 0.00097 | 0.00060 | 1064 | 1.60 | 0.1101 |
| task period Delay 1 | -0.00129 | 0.00060 | 1064 | -2.13 | 0.0331 * |
| task period Delay 2 | -0.00006 | 0.00060 | 1064 | -0.09 | 0.9252 |
| condition Diff | -0.00049 | 0.00060 | 1064 | -0.81 | 0.4154 |
| task period Encoding : condition Diff | 0.00058 | 0.00085 | 1064 | 0.67 | 0.5005 |
| task period Delay 1 : condition Diff | 0.00130 | 0.00085 | 1064 | 1.52 | 0.1279 |
| task period Delay 2 : condition Diff | -0.00024 | 0.00085 | 1064 | -0.28 | 0.7820 |

**IPL-HIP, 9-11 Hz**

| **fixed effect** | **Estimate** | **SE** | **DF** | **tStat** | **pValue** |
|---|---|---|---|---|---|
| intercept | -0.00168 | 0.00087 | 1064 | -1.93 | 0.0539 |
| task period Encoding | 0.00083 | 0.00043 | 1064 | 1.92 | 0.0555 |
| task period Delay 1 | -0.00094 | 0.00043 | 1064 | -2.17 | 0.0301 ***** |
| task period Delay 2 | -0.00084 | 0.00043 | 1064 | -1.93 | 0.0534 |
| condition Diff | 0.00019 | 0.00043 | 1064 | 0.43 | 0.6682 |
| task period Encoding : condition Diff | -0.00057 | 0.00061 | 1064 | -0.94 | 0.3478 |
| task period Delay 1 : condition Diff | -0.00020 | 0.00061 | 1064 | -0.33 | 0.7444 |
| task period Delay 2 : condition Diff | 0.00011 | 0.00061 | 1064 | 0.18 | 0.8584 |
